# Supplementary material for: A Comprehensive Assessment of Ultraviolet-Radiation-Induced Mutations in Flammulina filiformis Using Whole-Genome Resequencing
Source: J Fungi (Basel). 2024 Mar 20;10(3):228. doi: 10.3390/jof10030228 (PMC10971301; doi:10.3390/jof10030228)
Supplement: Supplementary file 1 [file jof-10-00228-s001.zip › Supplementary Material S8/KEGG annotation/out/64381550635650.os/KO/out_map/map04910.html]

KEGG PATHWAY: Insulin signaling pathway - Reference pathway


|  |  |
| --- | --- |
| **Insulin signaling pathway - Reference pathway** |  |

[
Pathway menu
| Organism menu
| Pathway entry
| Show description
| User data mapping
]

Insulin binding to its receptor results in the tyrosine phosphorylation of insulin receptor substrates (IRS) by the insulin receptor tyrosine kinase (INSR). This allows association of IRSs with the regulatory subunit of phosphoinositide 3-kinase (PI3K). PI3K activates 3-phosphoinositide-dependent protein kinase 1 (PDK1), which activates Akt, a serine kinase. Akt in turn deactivates glycogen synthase kinase 3 (GSK-3), leading to activation of glycogen synthase (GYS) and thus glycogen synthesis. Activation of Akt also results in the translocation of GLUT4 vesicles from their intracellular pool to the plasma membrane, where they allow uptake of glucose into the cell. Akt also leads to mTOR-mediated activation of protein synthesis by eIF4 and p70S6K. The translocation of GLUT4 protein is also elicited through the CAP/Cbl/TC10 pathway, once Cbl is phosphorylated by INSR.
Other signal transduction proteins interact with IRS including GRB2. GRB2 is part of the cascade including SOS, RAS, RAF and MEK that leads to activation of mitogen-activated protein kinase (MAPK) and mitogenic responses in the form of gene transcription. SHC is another substrate of INSR. When tyrosine phosphorylated, SHC associates with GRB2 and can thus activate the RAS/MAPK pathway independently of IRS-1.

|  |  |
| --- | --- |
| Reference pathway | 100% |
